# Disease burden and risk factors of ischemic heart disease in China during 1990–2019 based on the Global Burden of Disease 2019 report: A systematic analysis

**DOI:** 10.3389/fpubh.2022.973317

**Published:** 2022-11-03

**Authors:** Yanting Li, Jun Zhang

**Affiliations:** Department of Endocrinology and General Medicine, The First Hospital of Fangshan District, Beijing, China

**Keywords:** ischemic heart disease, China, disease burden, risk factors, Global Burden of Disease

## Abstract

**Objective:**

The aim of this study was to identify the disease burden and risk factors of ischemic heart disease (IHD) in China, during 1990–2019, through a systematic analysis using the Global Burden of Disease (GBD) 2019 report in order to provide first-hand information for primary and secondary prevention of IHD in China.

**Methods:**

Data on the rates of incidence, death, years of life lost (YLLs), years lived with disability (YLDs), and disability-adjusted life years (DALYs) of IHD were obtained from GBD2019 to determine the disease burden and risk factors of IHD in China.

**Results:**

The rates of incidence, death, YLLs, YLDs, and DALYs of IHD in China increased at different levels during 1990–2019. The annual rate of change in incidence, death, YLLs, YLDs, and DALYs of IHD were 1.31%, 1.57%, 0.93%, 1.14%, and 0.94%, respectively. In 2019, the YLDs of IHD in Chinese women were higher, while the rates of incidence and death, YLLs, and DALYs were lower in Chinese women than in Chinese men. The disease burden of IHD had significant age differences, and people aged ≥70 years had the highest disease burden. A total of 24 risk factors were associated with the rates of death and DALYs of IHD, and the five most significant risk factors were high systolic blood pressure, high LDL cholesterol (LDL-C), smoking, ambient particulate matter pollution, and intake of a high-sodium diet. From 1990 to 2019, a high annual rate of change in IHD-related deaths and DALYs was observed due to ambient particulate matter pollution, high body mass index (BMI), and intake of a diet high in processed meat.

**Conclusion:**

The results of the study revealed that the disease burden of IHD in China was on the rise, especially in people aged ≥70 years. The main disease burden of IHD in male patients was premature death and that in female patients was disability. Environmental, behavioral, and metabolic factors were considered the three main risks of the disease burden of IHD, with metabolic factors having the greatest impact. Therefore, periodic health check-ups and high-risk factor interventions for key populations should be strengthened from the grassroots level, which are conducive to further reducing the disease burden of IHD in China.

## Introduction

Ischemic heart disease (IHD), also referred to as coronary atherosclerotic heart disease, is a major chronic and fatal non-communicable disease, but the preferred management approach for this disease has not been well-defined (1, 2). Ischemic heart disease is one of the major contributors to cardiovascular disease (CVD)-related disease burden, which has been proven to be the leading cause of death in both developed and developing countries (3). Based on the Global Burden of Disease (GBD) 2019 report, the total number of deaths due to IHD reached 9.14 million in 2019 worldwide, accounting for 49.2% of CVD-related deaths (4). Except for life metrics such as disability-adjusted life years (DALYs), IHD has an enormous impact on productive life years (PLYs). Researchers projected that, by 2030, 8,100 people aged 45–64 years will be out of the labor force due to IHD in Australia, and there will be a 35% increase in government's welfare payments (5).

Currently, IHD has become a serious public health problem in China (6–8). In China, from 1990 to 2019, with the rapid development of the economy and social background, food consumption patterns, and lifestyles of people have changed dramatically. The most common feature is that the plant-based Eastern diet is gradually replaced by the unhealthy Western type of diet characterized by animal-based foods and foods with high added sugar (9). It has been reported that many known controllable risk factors contribute to IHD, such as hypertension, smoking, high LDL cholesterol (LDL-C), excessive sodium intake, and insufficient intake of nuts and seeds (3). Moreover, a survey conducted in the Hexi Corridor, Northwest China, showed that sandstorm weather is a risk factor for mortality in patients with IHD (10). In pursuit of better primary and secondary prevention of IHD, in this study, the current status and changing trends for the disease burden and risk factors of IHD in China were analyzed by using the GBD2019 data. The IHD-related rates of death and DALYs attributed to environmental, behavioral, and metabolic risk factors were assessed to propose more targeted strategies for IHD prevention in China.

## Methods

### Data sources

As a retrospective study, data were obtained from GBD2019, including the disease burden and risk factors of IHD in China. The GBD2019 database (http://ghdx.healthdata.org/gbdresults-tool) is created by the Institute for Health Measurement and Evaluation and the University of Washington, covering a total of 86,249 standardized data sources worldwide. The death data of various diseases were evaluated by coding the diseases according to the 10th edition of the International Classification of Diseases (ICD-10), and the coding range of IHD was I20–I25 (11). The death data of IHD in China were mainly derived from Disease Surveillance Points (DSPs), the Maternal and Child Surveillance System, and the Chinese Centers for Disease Control and Prevention's (CDC) cause-of-death-reporting system (12). Data on risk factors and disability disease burden were mainly derived from relevant monitoring and surveys over the years, as well as through a systematic review of relevant literature. Population data are mainly obtained from previous censuses (13). All the aforementioned data can be queried from the GBD Global Health Data Exchange Index (http://ghdx.healthdata.org/gbd-2019/data-input-sources; https://www.healthdata.org/).

### Risk factor classification

According to GBD2019, risk factors can be divided into environmental, behavioral, and metabolic risk factors. Environmental risk factors include ambient particulate matter pollution, household air pollution from solid fuels, and lead exposure. Behavioral risk factors include smoking; secondhand smoke; intake of a diet low in whole grains, vegetables, fruits, legumes, fiber, nuts and seeds, seafood omega-3 fatty acids, and polyunsaturated fatty acids; and intake of a diet high in processed meat, sugar-sweetened beverages, trans fatty acids, and sodium, as well as low physical activity and alcohol use. Metabolic risk factors include high systolic blood pressure, high fasting plasma glucose, high LDL-C, high body-mass index (BMI), and kidney dysfunction (14).

### Statistics

First, the rates of incidence, death, years of life lost (YLLs), years lived with disability (YLDs), and DALYs were used to describe the disease burden of IHD in different gender and age groups in China. Second, the death rate and DALYs attributed to 24 controllable risk factors for IHD were also analyzed. Finally, the changing trends in IHD-related risk factors from 1990 to 2019 were assessed with the annual rate of change caused by death and DALYs. Statistical analysis has been explained in detail in previous studies (15).

The rate of incidence can be defined as the frequency of new cases of a disease in a specified population within a given period. The rate of incidence can be used to reflect the effect of this disease on the population, which can be represented as follows: rate of incidence = the number of new cases/the number of exposures during the same period. The death rate is the ratio of the number of dead individuals in a specified period (usually 1 year) in a given region to the average population size during the same period represented as follows: Death rate = the number of dead individuals per unit time/average population size per unit time × 1,000%. YLLs represent years of life lost due to premature mortality, which is expressed as follows: YLLs = the longest possible life expectancy for a person in that age-group – the age at death. Years of life lost can also be described as years lived with any short- or long-term health loss, which can be expressed as follows: YLDs = prevalence × disability weights. Disability weights are derived from public surveys to reflect the severity of different cases. Disability-adjusted life years (DALYs) is the sum of YLLs and YLDs, and it is also a comprehensive indicator response to the number of lives and quality of life measured in time.

## Results

### Disease burden and risk factors of Chinese IHD in 2019

**Figure 1:**
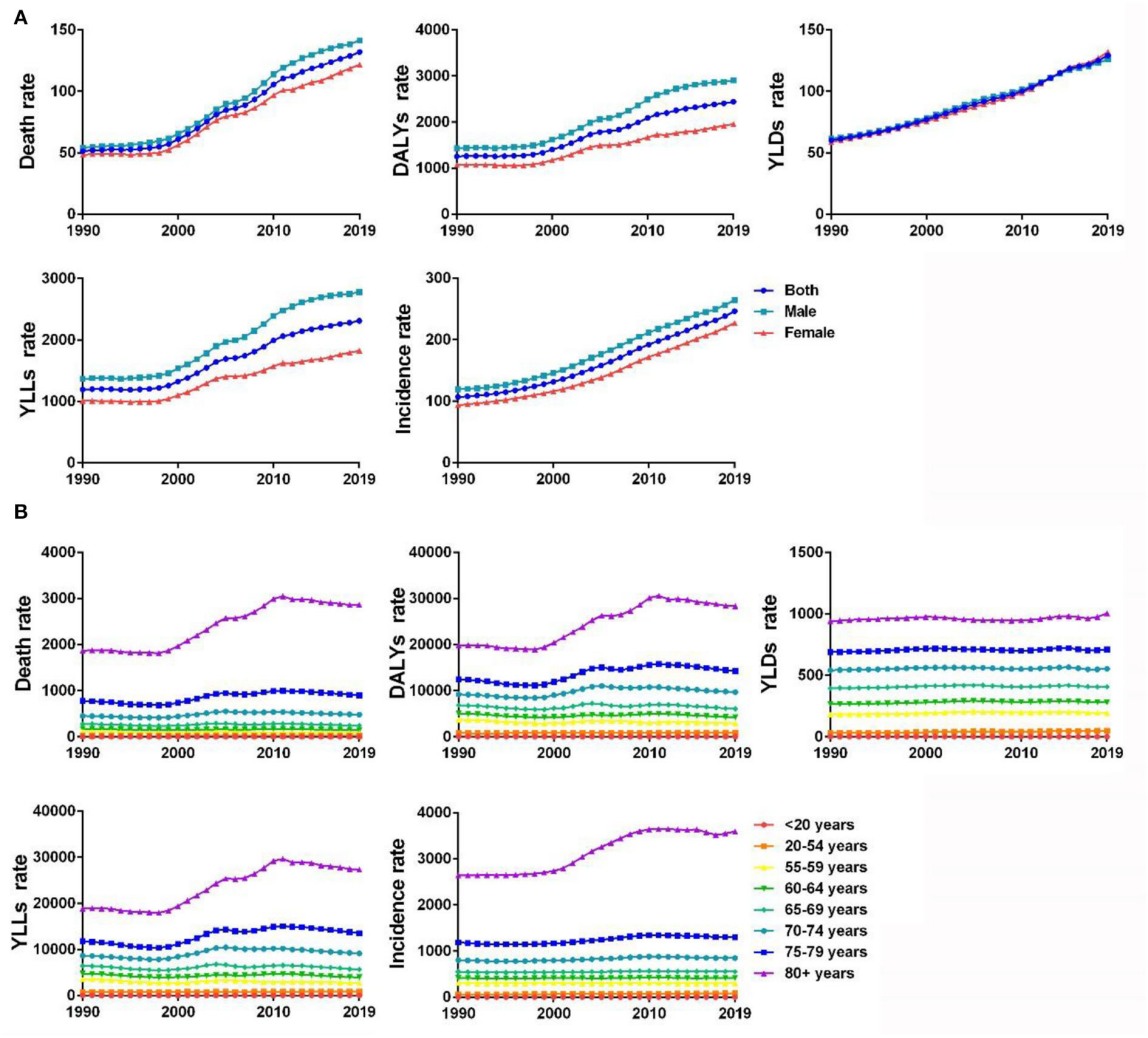
**(A)** Gender differences in disease burden of IHD in China from 1990 to 2019 (1/100,000). **(B)** Disease burden of IHD in different age-groups in China from 1990 to 2019 (1/100,000).

In 2019, the rates of incidence, death, YLLs, YLDs, and DALYs of IHD in the Chinese population were 246.06/100,000 [95% uncertainty interval (UI) 216.97–276.97], 131.75/100,000 (95% UI 113.34–149.88), 2309.58/100,000 (95% UI 2249.30–3392.04), 129.05/100,000 (95% UI 85.74–183.36), and 2438.63/100,000 (95% UI 2106.15–2786.90), respectively (Table 1 and Figure 1A). All index values increased with age, with an increase in people aged ≥70 years and a peak reached in people aged ≥80 years. The rates of incidence, death, YLLs, YLDs, and DALYs in people aged ≥80 years were 3592.57/100,000 (95% UI 2931.42–4404.11), 2860.87/100,000 (95% UI 2465.16–3205.22), 27314.99/100,000 (95% UI 23602.02–30715.79), 1002.59/100,000 (95% UI 643.34–1457.40), and 28317.57/100,000 (95% UI 24571.57–31660.46), respectively (Table 1 and Figure 1B).

**Table 1:** Disease burden of IHD in various age-groups in China, in 2019 (1/100,000).

**Age (years)**	**Incidence**	**Death**	**YLLs**	**YLDs**	**DALYs**
< 20	0.86 (0.16–1.93)	0.26 (0.22–0.31)	18.61 (15.43–22.29)	0.07 (0.03–0.15)	18.68 (15.51–22.36)
20–54	80.93 (65.17–99.33)	19.59 (16.14–23.58)	845.47 (698.09–1016.56)	47.84 (29.38–73.73)	893.31 (743.15–21062.71)
55–59	298.50 (205.71–409.16)	82.74 (68.04–99.86)	2682.20 (2205.69–3232.27)	192.43 (118.59–301.76)	2874.63 (2382.08–3435.54)
60–64	412.90 (319.82–523.79)	141.96 (118.01–167.92)	3950.34 (3283.65–4673.16)	278.96 (170.87–430.06)	4229.30 (3549.57–4971.77)
65–69	550.11 (398.74–709.96)	242.19 (204.88–282.77)	5644.85 (4775.18–6591.11)	406.07 (254.71–590.58)	6050.92 (5178.83–6993.22)
70–74	844.76 (670.85–1013.78)	478.36 (411.70–551.14)	9122.24 (7850.92–10508.92)	552.05 (351.14–795.75)	9674.29 (8361.26–11032.98)
75–79	1299.53 (942.76–1780.48)	894.94 (768.15–1018.89)	13491.69 (11576.89–15363.33)	708.70 (441.80–1036.23)	14200.39 (12311.03–16045.1)
≥80	3592.57 (2931.42–4404.11)	2860.87 (2465.16–3205.22)	27314.99 (23602.02–30715.79)	1002.59 (643.34–1457.40)	28317.57 (24571.57–31660.46)
Total	246.09 (216.97–276.97)	131.75 (113.34–149.88)	2309.58 (1974.13–2653.03)	129.05 (85.74–183.36)	2438.63 (2106.15–2786.90)

YLLs, years of life lost; YLDs, years lived with disability; DALYs, disability-adjusted life years.

In 2019, the rate of YLDs of IHD in Chinese women [132.08/100,000 (95% UI 87.75–186.90)] were higher than those in their male counterparts [126.12/100,000 (95% UI 83.29–182.24)], while the rate of incidence [227.03/100,000 (95% UI 200.23–256.23)], the rate of death [121.64/100,000 (95% UI 98.60–145.14)], YLLs [1823.52/100,000 (95% UI 1471.67–2216.15)], and the rate of DALYs [1955.61/100,000 (95% UI 1601.81–2336.10)] in women were lower than those in their male counterparts (Table 2 and Figure 1A).

**Table 2:** Gender differences in disease burden of IHD in China in 2019 (1/100,000).

**Age (years)**	**Incidence**	**Death**	**YLLs**	**YLDs**	**DALYs**
Male	264.44 (233.12–297.94)	141.49 (116.82–168.64)	2777.33 (2249.30–3392.04)	126.12 (83.29–182.24)	2903.46 (2378.47–3523.90)
Female	227.03 (200.23–256.23)	121.64 (98.60–145.14)	1823.52 (1471.67–2216.15)	132.08 (87.75–186.90)	1955.61 (1601.81–2336.10)
Total	246.09 (216.97–276.97)	131.75 (113.34–149.88)	2309.58 (1974.13–2653.03)	129.05 (85.74–183.36)	2438.63 (2106.15–2786.90)

Among the controllable risk factors for IHD in 2019, the metabolic risk was the primary risk factor leading to IHD-related death [103.34/100,000 (95% UI 86.41–120.40)] and DALYs [1972.50/100,000 (95% UI 1668.85–2292.02)] in China. However, the impact of environmental and behavioral risk factors on IHD also cannot be ignored. The death rates of IHD caused by environmental and behavioral risk factors were [45.79/100,000 (95% UI 37.90–53.52)] and [87.17/100,000 (95% UI 72.46–102.61)] in China. The rate of DALYs of IHD caused by both environmental and behavioral risk factors were [926.40/100,000 (95% UI 775.96–1088.77)] and [1741.03/100,000 (95% UI 1464.22–2059.41)]. Compared with the global trend, the death rates of the three risk factors have increased in China. For DALYs of IHD, China had a higher impact on environmental and behavioral risk factors but a lower impact on the metabolic risk factors than the global trend (Table 3). Gender and age differences in controllable risk factors were explored in China. The impact of the controllable risk factors on the IHD-related death rate and DALYs in men was higher than that in women, with an age-dependent overall increase (Table 3).

**Table 3:** Death rate and DALYs of risk factor-related IHD in China and worldwide in 2019 (1/100,000).

		**China**		**Global**	
**Risk factors**		**Death**	**DALYs**	**Death**	**DALYs**
Environmental factors	Total	45.79 (37.90–53.52)	926.40 (775.96–1088.77)	33.81 (29.68–37.99)	767.50 (674.44–860.76)
Behavioral factors	Total	87.17 (72.46–102.61)	1741.03 (1464.22–2059.41)	76.21 (66.82–85.71)	1627.07 (1448.62–1803.59)
Metabolic factors	Total	103.34 (86.41–120.40)	1972.50 (1668.85–2292.02)	97.28 (86.04–107.42)	1979.10 (1793.06–2155.69)
Sex
Environmental factors	Male	51.88 (41.53–62.59)	1149.33 (914.00–1403.05)	39.13 (34.01–44.22)	966.33 (845.18–1090.32)
	Female	39.46 (31.43–48.55)	694.75 (553.91–853.57)	28.46 (23.95–32.83)	567.41 (486.56–643.70)
Behavioral factors	Male	101.90 (82.99–123.36)	2239.89 (1821.70–2735.89)	88.49 (78.55–98.48)	2094.56 (1877.80–2317.86)
	Female	71.86 (54.89–89.83)	1222.66 (945.00–1534.67)	63.85 (53.99–74.20)	1156.62 (996.21–1322.36)
Metabolic factors	Male	110.98 (89.13–134.20)	2353.89 (1884.46–2855.03)	105.41 (94.22–116.20)	2404.18 (2171.13–2635.87)
	Female	95.40 (75.29–117.03)	1576.19 (1263.85–1916.14)	89.10 (76.60–100.59)	1551.32 (1374.42–1716.45)
Age
Environmental factors	< 20	0.02 (0.01–0.03)	1.50 (1.00–2.04)	0.02 (0.01–0.03)	1.37 (0.76–1.84)
	20–54	9.22 (7.39–11.29)	418.27 (340.85–502.39)	11.33 (9.79–12.93)	504.00 (436.89–571.40)
	55–59	36.54 (28.61–45.62)	1259.01 (993.80–1562.09)	55.64 (46.34–65.57)	1847.85 (1536.83–2177.29)
	60–64	59.41 (46.54–73.58)	1754.29 (1390.26–2145.18)	81.97 (67.83–96.36)	2341.73 (1929.60–2754.58)
	65–69	96.31 (76.50–116.97)	2382.86 (1908.88–2878.55)	116.58 (97.59–135.44)	2808.52 (2352.93–3270.92)
	70–74	178.84 (142.76–217.00)	3583.98 (2878.80–4327.81)	172.02 (142.29–203.32)	3391.24 (2819.32–4008.56)
	75–79	310.22 (252.03–371.44)	4879.70 (3977.65–5853.99)	256.28 (211.42–300.34)	3988.22 (3298.89–4684.98)
	≥80	835.39 (687.47–981.87)	8352.89 (6883.06–9843.81)	520.71 (426.34–616.40)	5075.26 (4187.78–5985.60)
Behavioral factors	< 20	−0.00 (−0.01 to −0.00)	−0.27 (−0.49 to −0.08)	−0.00 (−0.01 to −0.00)	−0.23 (−0.37 to −0.07)
	20–54	16.78 (13.76–20.39)	757.96 (628.84–915.57)	21.81 (19.63–23.96)	962.37 (866.02–1056.68)
	55–59	68.03 (54.67–82.91)	2355.04 (1914.90–2867.23)	114.18 (102.37–125.78)	3802.13 (3414.35–4190.16)
	60–64	112.97 (92.81–137.03)	3354.40 (2767.52–4037.35)	175.85 (157.73–193.91)	5040.73 (4522.85–5563.22)
	65–69	179.54 (147.24–213.89)	4471.68 (3698.05–5321.90)	245.30 (217.45–273.11)	5941.17 (5251.17–6636.79)
	70–74	334.06 (274.75–398.55)	6737.98 (5556.98–7961.62)	368.35 (325.38–412.65)	7312.17 (6453.34–8183.30)
	75–79	565.94 (465.34–675.20)	8960.71 (7319.75–10611.32)	550.02 (476.85–622.63)	8624.70 (7480.91–9793.45)
	≥80	1659.36 (1341.78–1970.60)	16548.56 (13508.20–19651.93)	1393.07 (1140.31–1623.50)	13325.98 (11099.37–15460. 29)
Metabolic factors	< 20	0.00 (0.00–0.00)	0.00 (0.00–0.00)	0.00 (0.00–0.00)	0.00 (0.00–0.00)
	20–54	17.46 (14.24–21.16)	793.61 (653.05–946.70)	24.06 (21.67–26.47)	1064.74 (958.99–1173.32)
	55–59	72.19 (57.99–87.64)	2508.35 (2027.43–3007.77)	132.56 (120.31–144.15)	4416.04 (4004.15–4796.73)
	60–64	120.03 (97.38–145.29)	3576.29 (2924.14–4296.55)	207.25 (189.61–225.58)	5942.72 (5447.08–6464.11)
	65–69	195.13 (157.90–234.64)	4875.19 (4002.01–5800.24)	298.69 (269.19–328.98)	7235.87 (6501.62–7995.35)
	70–74	370.95 (298.10–447.04)	7501.90 (6061.51–9050.98)	456.40 (402.95–510.01)	9064.12 (8044.83–10122.94)
	75–79	696.70 (579.80–821.19)	11055.04 (9264.58–12968.45)	751.17 (665.73–828.34)	11786.17 (10432.58–13007.21)
	≥80	2147.08 (1691.02–2566.07)	21248.32 (16919.75–25216.83)	1941.91 (1561.35–2256.69)	18442.87 (15020.66–21345.93)

**Figure 2:**
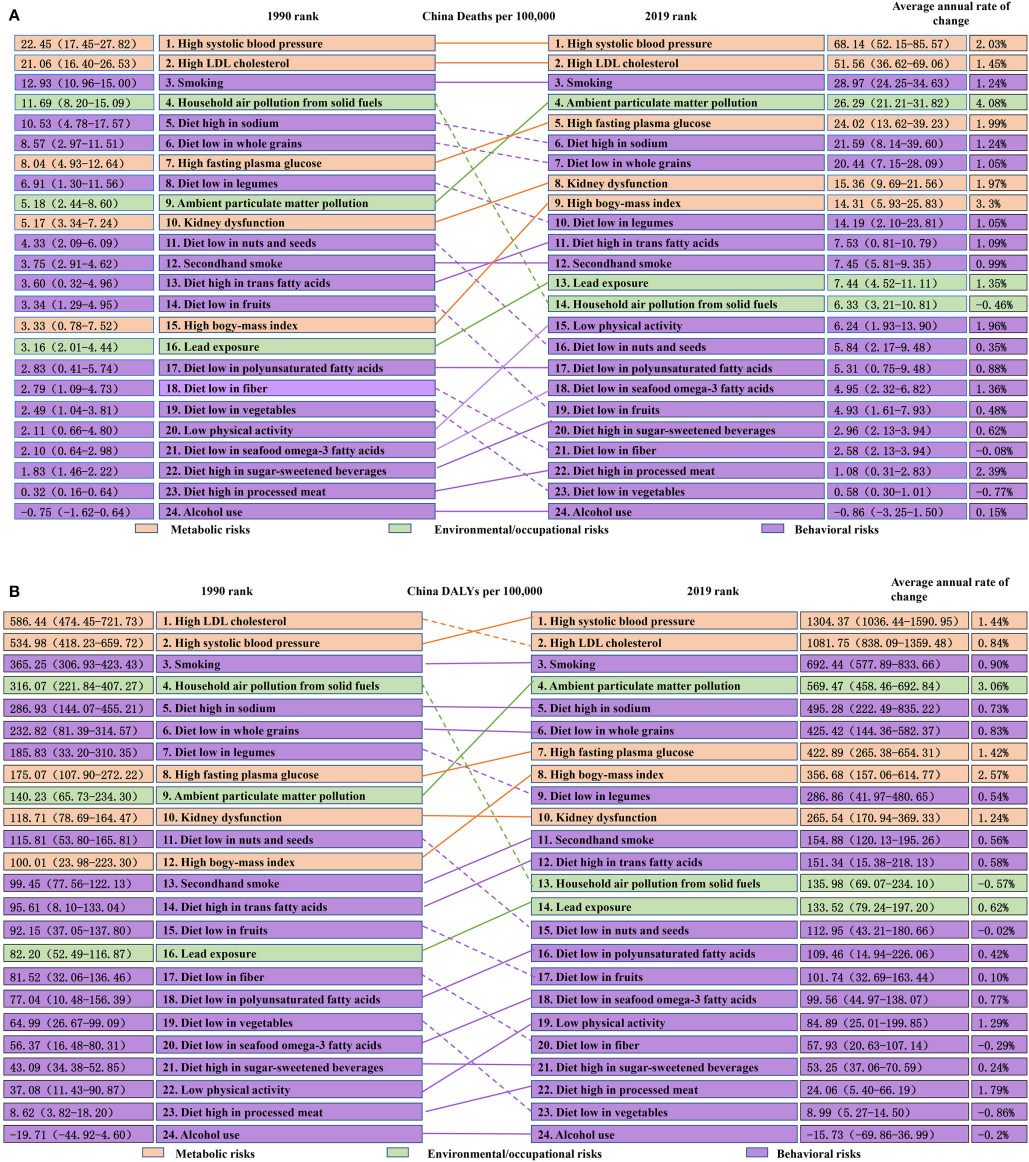
**(A)** Death rate of risk factor-related IHD in China in 1990 and 2019 (1/100,000). **(B)** DALYs of risk factor-related IHD in China in 1990 and 2019 (1/100,000).

As shown in Figure 2A, the five most important risk factors associated with the death of IHD in China were high systolic blood pressure [68.14/100,000 (95% UI 52.15–85.57)], high LDL-C [51.56/100,000 (95% UI 36.62–69.06)], smoking [28.97/100,000 (95% UI 24.25–34.63)], ambient particulate matter pollution [26.29/100,000 (95% UI 21.20–31.82)], and high fasting blood glucose [24.02/100,000 (95% UI 13.62–39.23)]. The five most important risk factors influencing DALYs of IHD in China were high systolic blood pressure [1304.37/100,000 (95% UI 1036.44–1590.95)], high LDL-C [1081.75/100,000 (95% UI 838.09–1359.48)], smoking [692.44/100,000 (95% UI 577.89–833.66)], ambient particulate matter pollution [569.47/100,000 (95% UI 458.46–692.84)], and intake of a high-sodium diet [495.28/100,000 (95% UI 222.49–35.22)] (Figure 2B).

### Change trends of disease burden and risk factors of IHD in China from 1990 to 2019

From 1990 to 2019, the rates of incidence, death, YLLs, YLDs, and DALYs of IHD in China increased at different levels. The rate of incidence increased from 106.64/100,000 (95% UI 93.87–120.06) in 1990 to 246.09/100,000 (95% UI 216.97–276.97) in 2019, with an annual rate of change of 1.31% (95% UI 1.22–1.40%). The rate of death increased from 51.34/100,000 (95% UI 45.31–257.34) in 1990 to 131.75/100,000 (95% UI 113.34–149.88) in 2019, with an annual rate of change of 1.57% (95% UI 1.16–2.06%). The rate of YLLs increased from 1193.91/100,000 (95% UI 1042.86–1350.43) in 1990 to 2309.58/100,000 (95% UI 2249.30–3392.04) in 2019, with an annual rate of change of 0.93% (95% UI 0.59–1.37%). The rate of YLDs increased from 60.18/100,000 (95% UI 40.04–85.99) in 1990 to 129.05/100,000 (95% UI 85.74–183.36) in 2019, with an annual rate of change of 1.14% (95% UI 1.09–1.20%). The rate of DALYs increased from 1254.09/100,000 (95% UI 1096.61–1408.23) in 1990 to 2438.63/100,000 (95% UI 2106.15–2786.90) in 2019, with an annual rate of change of 0.94% (95% UI 0.62–1.36%) (Table 4). From 1990 to 2019, the rates of incidence, death, YLLs, YLDs, and DALYs of IHD in men and women showed an increasing trend. The annual rate of change for IHD-related death [1.43% (95% UI 1.34–1.54%)] and YLDs [1.25% (95% UI 1.19–1.31%)] in women were higher than those in men [1.22% (95% UI 1.13–1.32%) and 1.05% (95% UI 0.98–1.12%), respectively] (Table 5). The rates of death, DALYs, and YLLs of IHD in the Chinese population aged 20–54 years and ≥70 years, as well as the incidence rate of IHD in people aged 20–54 years and ≥65 years and the rate of YLDs of IHD in people aged ≥20 years, were higher in 2019 than in 1990 (Figure 1B).

**Table 4:** Disease burden (1/100,000) and annual rate of change of IHD in China from 1990 to 2019.

**Year**	**Incidence**	**Death**	**YLLs**	**YLDs**	**DALYs**
1990	106.64 (93.87–120.06)	51.34 (45.31–257.34)	1193.91 (1042.86–1350.43)	60.18 (40.04–85.99)	1254.09 (1096.61–1408.23)
2019	246.09 (216.97–276.97)	131.75 (113.34–149.88)	2309.58 (2249.30–3392.04)	129.05 (85.74–183.36)	2438.63 (2106.15–2786.90)
1990–2019	1.31% (1.22–1.40%)	1.57% (1.16–2.06%)	0.93% (0.59–1.37%)	1.14% (1.09–1.20%)	0.94% (0.62–1.36%)

**Table 5:** Annual rate of change of gender-specific disease burden of IHD in China from 1990 to 2019.

**Sex**	**Incidence**	**Death**	**YLLs**	**YLDs**	**DALYs**
Male	1.22% (1.13–1.32%)	1.62% (1.06–2.38%)	1.03 (0.54–1.17%)	1.05% (0.98–1.12%)	1.03% (0.56–1.68%)
Female	1.43% (1.34–1.54%)	1.51% (0.95–2.17%)	0.81% (0.38–1.34%)	1.25% (1.19–1.31%)	0.83% (0.43–1.33%)

As shown in Figure 2A, from 1990 to 2019 in China, the annual rate of change of five metabolic risk factors for IHD-related death in China increased, with high BMI (3.3%), high systolic blood pressure (2.03%), and high fasting blood glucose (1.99%) showing a higher annual rate of change. As for the 16 behavioral risk factors for death caused by IHD, the annual rate of change due to intake of a diet low in vegetables (−0.77%) and fiber (−0.08%) decreased, whereas the remaining 14 behavioral risk factors led to an increase in the annual rate of change and an intake of a diet high in processed meat (2.39%), low physical activity (1.96%), and intake of a diet low in seafood omega-3 fatty acids (1.36%) should be of particular concern. With regard to environmental risk factors for IHD-related death, household air pollution from solid fuels (−0.46%) led to a decrease in the annual rate of change, inconsistent with ambient particulate matter pollution and lead exposure, and the focus should be given on ambient particulate matter pollution (4.08%) with a relatively large change.

Analysis of the annual rate of change for IHD-related DALYs in China during 1990–2019 showed the annual rate of change due to the five controllable metabolic risk factors has increased, with high BMI (2.57%), high systolic blood pressure (1.44%), and high fasting blood glucose (1.42%) showing a greater annual rate of change. As for the 16 controllable behavioral risk factors for IHD-related DALYs, the annual rate of change for intake of a diet low in vegetables (−0.86%) and fiber (−0.29%), alcohol use (−0.20%), and intake of a diet low in nuts and seeds (−0.02%) has declined, whereas the opposite trend was reported for the 12 remaining behavioral risk factors. A diet high in processed meat (1.79%), low physical activity (1.29%), and smoking (0.90%) had the most significant increases in the annual rate of change. We finally analyzed three controllable environmental risk factors of IHD-related DALYs. The annual rate of change due to household air pollution from solid fuels (−0.57) has decreased, but the annual rate of change due to ambient particulate matter pollution and lead exposure has increased, and the annual rate of change due to ambient particulate matter pollution (3.06%) should be of particular concern (Figure 2B).

## Discussion

Ischemic heart disease refers to the stenosis or occlusion of the coronary artery caused by atherosclerosis, resulting in myocardial hypoxia or necrosis-induced heart disease, which seriously endangers human health. In 2019, 61.00% of the health burden of cardiovascular disease in China was found to be caused by atherosclerotic cardiovascular disease (ASCVD), in which IHD ranked second after ischemic stroke (13). In 2019, the rate of YLLs of IHD was 2309.58/100,000, which was 17.89 times higher than the rate of YLDs, indicating that premature death was the main cause of the disease burden of IHD in China.

In this report, gender differences were found in the disease burden of IHD in China. The rates of incidence, death, and DALYs of IHD in Chinese men were higher than those in women, but the annual rate of change of IHD in death and YLDs in were higher in women than in men. DALYs of IHD was mainly caused by premature death in male patients, but disability in female patients. In addition, the impact of the controllable risk factors on IHD-related death rate and DALYs in men was higher than that in women. The reasons for the higher disease burden of IHD in Chinese men than women may be heavy social responsibilities and great mental/psychological pressure faced by men, making them prone to developing unhealthy living habits such as staying up late, smoking, excessive drinking, and intake of a high-fat diet. Compared with women, men are more likely to ignore their health problems and fail to seek medical treatment in time. Moreover, the protective effect of estrogen in women can prevent diabetes, protect blood vessels, and delay atherosclerosis; however, the extent of the protective effect needs to be supported by further evidence (16).

With the growth of the population and the intensification of population aging, the absolute number of IHD deaths is continuously increasing. Furthermore, the incidence of chronic diseases such as hypertension and diabetes are on the rise. The improvement of the healthcare level has shown to prolong the survival period of patients with heart disease. It can be inferred from the aforementioned results that the incidence and severity of IHD increase with age (17). Our study also found age differences in the disease burden of IHD in China, and the overall trend of the disease burden of IHD due to controllable risk factors increases with age. This study showed that from 1990 to 2019, the disease burden of IHD in Chinese people aged ≥70 years has significantly increased, with a peak in people aged ≥80 years. The death rate and DALYs of IHD in the Chinese population aged 20–54 years were higher in 2019 than in 1990, which is consistent with previous research findings that the rates of IHD incidence and death in China show an increasing trend in younger people (18, 19) as a result of overweight, smoking, abnormal lipid metabolism, high blood uric acid, inflammatory response, and impaired vascular endothelial function in young and middle-aged people (20).

In addition to the influence of gender and age, controllable factors such as hypertension, dyslipidemia, diabetes, smoking, and obesity are also closely related to the disease burden of IHD (21). In this study, GBD2019 data were used to estimate the changing trends of 24 risk factors attributable to IHD in the Chinese population, which were divided into three categories: environmental, behavioral, and metabolic factors. Our results showed that metabolic risk factors had the greatest impact on IHD death rate and DALYs in China, followed by environmental and behavioral risk factors. Compared with the global trends, the three major risk factors had a higher impact on the death rate of IHD in China. The impact of environmental and behavioral risk factors on DALYs of IHD in China was higher and that of metabolic risk factors was lower than the global trend. China is a country with a large population and is a developing country, with a relatively low economy. Moreover, in China, the rapid development of the economy has resulted in environmental damage, and the unbalanced development of education has resulted in the insufficient awareness of diseases and behavioral restraint. Furthermore, with the development of the economy, the diet structure of Chinese people has changed, characterized by increased intake of protein and fat and insufficient intake of cereals and vegetables, which resulted in a higher death rate of IHD in China than globally. However, the diet structure of people in developed countries is more inclined to high-fat and high-protein diets, so the DALYs of IHD caused by metabolic risk factors in China is slightly lower than that globally. This study reported that among the 24 controllable risk factors, the five most influencing factors were high systolic blood pressure, high LDL-C, smoking, ambient particulate matter pollution, and intake of a high-sodium diet. Blood pressure is significantly related to the progression of IHD. The higher the blood pressure, the longer the course of the disease, and the easier it is to develop multivessel, diffuse, and complex coronary vascular disease (22). High LDL-C can damage the endothelial cell structure and lead to lipid infiltration and the formation of atherosclerosis. The rupture of atherosclerotic plaques can lead to the formation of microthrombi, which is a risk factor for IHD (23). Smoking can damage the vascular endothelium, promote platelet adhesion and aggregation, increase thrombosis, narrow the arterial lumen, block arterial blood flow, and eventually lead to a variety of cardiovascular and cerebrovascular diseases (24). Studies have shown that there is a correlation between ambient particulate matter pollution and IHD deaths (25). A high-sodium diet can cause water and sodium retention, predispose to high blood pressure, and increase the risk of IHD. In this study, from 1990 to 2019, five controllable metabolic risk factors have led to an increase in the annual rate of change of IHD-related death and DALYs, with high BMI, high systolic blood pressure, and high fasting blood glucose showing the highest change. In the 16 controllable behavioral risk factors for IHD-related death, the annual rate of change caused by intake of a diet low in vegetables and fiber decreased, whereas that of the remaining 14 behavioral risk factors showed increased, with high intake of processed meat, low physical activity, and intake of seafood low in omega-3 fatty acids showing the most obvious change. The annual rates of change in DALYs for intake of a diet low in vegetables, fiber, and nuts and seeds, and alcohol use were decreased, whereas those of the remaining 12 behavioral risk factors increased, with intake of a diet high in processed meat, low physical activity, and smoking showing the largest annual changes. The improved lifestyle and accelerated life rhythm changed Chinese diet structure. Being overweight is a consequence of preferring a diet high in processed meat diet and lack of physical activity. Being overweight and smoking increased the incidence of IHD, which can further lead to high risk for recurrent events of kidney dysfunction (26). Moreover, in China, the rapid development of economy has inevitably aggravated environmental damage. For the three controllable environmental risk factors for IHD-related death rate and DALYs, the annual rate of change caused by household air pollution from solid fuels decreased in contrary to the other two environmental risk factors. Among them, the annual rate of change in the disease burden of IHD caused by ambient particulate matter pollution was relatively large (24). However, with the improvement of living standards and educational levels, Chinese people pay more attention to their health and also increase the intake of vegetables and fiber. In addition, the using of natural gas and central heating reduce the environmental pollution caused by the household air pollution from solid fuels. The aforementioned positive changes may have a positive impact on the primary and secondary prevention of IHD in China.

## Conclusion

Given that China is a country with a large aging population and that its medical resources are in short supply, periodic health check-ups and high-risk factor interventions for key populations should be reinforced from the grassroot level. Based on GBD2019 data, the burden and risk factors of IHD in China were analyzed, which not only provide a reference for IHD prevention formulated by public policymakers but also alert patients by increasing health education. According to our analytical data, the following healthy lifestyles can help strengthen IHD management and prevention: health lectures, spreading awareness of the danger of smoking, 150 min of aerobic exercise per week, improvement of the unreasonable diet structure, and promoting trams and bicycles to reduce environmental pollution. Moreover, due to the increase in the incidence of IHD in young and middle-aged people, health education for these populations could help improve patient compliance with treatment, such as informing about reasonable diet, exercise, and weight loss, limiting smoking and alcohol, and reducing the disease burden of IHD in China.

## Data availability statement

The original contributions presented in the study are included in the article/supplementary material, further inquiries can be directed to the corresponding author/s.

## Ethics statement

Ethical review and approval was not required for the study on human participants in accordance with the local legislation and institutional requirements. Written informed consent for participation was not required for this study in accordance with the national legislation and the institutional requirements.

## Author contributions

YL conceived the idea of the study, drafted the manuscript, and drew the figures. JZ reviewed and approved the submitted manuscript version. Both authors contributed to the article and approved the submitted version.

## Conflict of interest

The authors declare that the research was conducted in the absence of any commercial or financial relationships that could be construed as a potential conflict of interest.

## Publisher's note

All claims expressed in this article are solely those of the authors and do not necessarily represent those of their affiliated organizations, or those of the publisher, the editors and the reviewers. Any product that may be evaluated in this article, or claim that may be made by its manufacturer, is not guaranteed or endorsed by the publisher.
